# Influence of Sugar-Sweetened Beverages Intake on Sarcopenic Obesity, Visceral Obesity, and Sarcopenia in Lebanese Patients with MASLD: A Case-Control Study

**DOI:** 10.3390/healthcare12050591

**Published:** 2024-03-05

**Authors:** Maha Hoteit, Myriam Dagher, Nikolaos Tzenios, Najat Al Kaaki, Ghadir Rkein, Abdul Rahman Chahine, Yonna Sacre, Samer Hotayt, Rami Matar, Mahmoud Hallal, Micheal Maitar, Bilal Hotayt

**Affiliations:** 1 Food Science Unit, National Council for Scientific Research-Lebanon (CNRS-Lebanon), Beirut P.O. Box 11-8281, Lebanon; 2 Faculty of Public Health, Lebanese University, Beirut P.O. Box 14-6573, Lebanon; nana.alkaaki@hotmail.com (N.A.K.); ghadirrkein12@hotmail.com (G.R.); 3 Faculty of Health Sciences, American University of Beirut, Beirut P.O. Box 11-0236, Lebanon; md133@aub.edu.lb; 4 Faculty of Public Health, Charisma University, London EC1V 7QE, UK; nicolas@trccolleges.com; 5 Radiology Department, Beirut Arab University, Beirut P.O. Box 11-5020, Lebanon; abderahmans@yahoo.com; 6 Department of Nutrition and Food Sciences, Faculty of Arts and Sciences, Holy Spirit University of Kaslik (USEK), Jounieh P.O. Box 446, Lebanon; 7 Anesthesia Department, Saint Joseph Hospital, 75014 Paris, France; samerhotayt@hotmail.com; 8 School of Medicine, St. George’s University, West Indies FZ818, Grenada; rmatar1@sgu.edu; 9 Gastroenterology Department, Faculty of Medical Science, Lebanese University, Beirut P.O. Box 14-6573, Lebanon; mahmoud.hallal@zhumc.org.lb; 10 Gastroenterology and Hepatology Department, Zahraa University Medical Center (ZHUMC), Beirut P.O. Box 90-361, Lebanon; 11 Division of Gastroenterology, Hepatology, and Nutrition, Department of Internal Medicine, Southern Illinois University, Springfield, IL 62901, USA; mmaitar25@siumed.edu; 12 Gastroenterology Department, Sahel General Hospital, Beirut P.O. Box 90-1603, Lebanon

**Keywords:** dysfunction-associated steatotic liver disease, sarcopenia, sarcopenic obesity, visceral obesity, Lebanon, sugar-sweetened beverages, nutrition

## Abstract

Chronic liver diseases are a major global health concern. Aims: this study investigated the links between medical, clinical, anthropometric, and dietary factors with dysfunction-associated steatotic liver disease (MASLD) in the Lebanese population using a case-control approach to uncover factors influencing visceral obesity, sarcopenia, and sarcopenic obesity. Methods and Materials: a total of 120 participants (20–70 years old) were divided into case and control groups based on liver disease diagnosis. Patient information was gathered through a questionnaire encompassing demographics, medical history, and beverage consumption. Anthropometric and body composition data were collected in a clinical setting. Results: our findings indicated a clear association between the presence of MASLD and obesity, hypertension, and diabetes. The positive association with higher body mass index and all three conditions remained consistent even when data was stratified by case and control groups. A greater proportion of MASLD patients exhibited sarcopenic obesity. Furthermore, MASLD cases showed higher consumption of sugary beverages and a reduced intake of milk and water in their diets. Conclusions: this study shed light on the health attributes and diets of the Lebanese population with liver diseases and suggested more research in this area and in a more ethnically diverse population.

## 1. Introduction

Chronic liver diseases have been recognized to be one of the leading causes of morbidity and mortality worldwide [1,2,3] The Global Burden of Disease provided evidence that dysfunction-associated steatotic liver disease (MASLD), is one of the most common culprits of chronic liver diseases with the most rapidly increasing burden [4,5]. MASLD includes a range of liver conditions in which the hepatic accumulation of lipids encompasses a wide spectrum of conditions, spanning from an early-stage steatosis known as non-alcoholic hepatic steatosis (NAFL) to a progressive form of non-alcoholic steatohepatitis (NASH) which can extend to advanced cirrhosis with its clinical consequences and can potentially develop into liver failure [6,7,8,9]. MASLD has been increasing over the last decade at an alarming rate with a recently estimated prevalence of 33%, making it a major public health problem worldwide [4,10,11,12]. Notably, MASLD is posing a substantial burden, particularly for low- and middle-income countries undergoing remarkable nutrition transition, including countries of the Middle East and North Africa (MENA) region. Lebanon, a small middle-income country in the MENA region, experienced recently a worsening trend in the death rate, with an incidence rate of liver complications due to MASLD per 100,000 almost reaching the global incidence rate [4]. Subsequently, liver-related morbidity and mortality in Lebanon and in the MENA are expected to rise rapidly in the future with the growing rates of obesity, metabolic syndrome, and its related comorbidities amidst the nutrition transition, all of which were shown to be strongly associated with MASLD [13,14]. The marked nutrition transition is characterized by a shift in dietary intake and food consumption behaviors leaning towards more highly processed, ‘westernized’ dietary patterns along with increased sedentary behaviors [15]. Notably, Lebanon has witnessed a concerning trend in the increased consumption of sugar-sweetened beverages especially amongst children and adolescents [15,16]. These trends have been contributing to the development and progression of overweight and obesity in the country, reaching 65% and 27%, respectively, among Lebanese aged 18–69 years in 2020 [17], reflecting these shifting dietary habits and lifestyle change, which is leading to metabolic and health consequences threatening the health of the population in the short and long run. Furthermore, metabolic syndrome has been shown to be closely associated with the development of liver fibrosis through various underlying mechanisms. Patients with more than one of the features of metabolic syndrome or type 2 diabetes are at increased risk of cirrhosis and related hepatic complications [18,19,20]. Furthermore, in recent years, age-related decreases in appendicular skeletal muscle mass, also known as sarcopenia, has been a growing area of interest. Cross-sectional studies have been seeing the manifestation of sarcopenia in the early stages of liver disease worsening its prognosis [21,22,23]. This association can be explained by underlying mechanisms like insulin resistance, metabolic syndrome, chronic inflammation, and changes in growth hormones, all of which play a key role in MASLD pathophysiology [2,19,22,24]. The coexistence of excess adiposity and reduced skeletal muscle mass is called sarcopenic obesity [25,26]. Sarcopenic obesity has been recently looked up as an additional key factor associated with a prognostic deterioration of liver diseases due to its cardiometabolic risks compared to obesity or sarcopenia alone [27,28,29]. There is a lack of research on the association of the overarching risk factors of MASLD among the MENA population. With the rise of obesity, sarcopenia, and sarcopenic obesity in Lebanon [15,16], it has become critical to study the risk factors related to the development and progression of MASLD. Thus, this study aimed to explore the association between dietary, medical, clinical, and anthropometric characteristics with MASLD and the factors affecting visceral obesity, sarcopenia, and sarcopenic obesity among the Lebanese population through a case-control design.

## 2. Subjects and Methods

### 2.1. Study Design and Subjects

This study followed a case-control design and included a total of 120 participants aged between 20 and 70 years old who were identified from a database of health centers, medical centers, private clinics, and hospitals of trusted gastroenterologists in Lebanon, for a duration of three months (March–June 2019). Cases were categorized based on medical diagnosis through the physician or the hospital diagnosis and included patients diagnosed with MASLD with alcohol consumption of less than 20 g/day in the year before diagnosis. Controls included patients free from MASLD who were identified from the same database, and whose age and sex matched with the cases. Exclusion criteria included pregnant women and patients with viral hepatitis or other forms of liver disease of different or mixed etiology as determined via serology and liver histology (excessive alcohol consumption, hepatitis C, hepatitis B, autoimmune liver disease, Wilson’s disease, hemochromatosis, alpha 1—antitrypsin deficiency, decompensated cirrhosis, jaundice, presence of ascites or encephalopathy, and hepatocellular carcinoma). Additional exclusion criteria were patients with cancer, HIV infection, active intravenous drug addiction, and regular use of steatosis-inducing drugs. Patients with diabetes type II were also excluded from the control group. The study was conducted according to the guidelines of the Declaration of Helsinki, and approved by the ethical committee of Al Zahraa University Medical Center #158/5 December 2018). Informed consent was obtained from all subjects involved in the study. Written informed consent was obtained from the patient(s) to publish this paper.

### 2.2. Questionnaire

The past and current history of patients was collected using a developed questionnaire. In brief, the questions raised revolved around three categories of data (i) demographics (age, gender, marital status) and self-reported height; (ii) current and past medical history; (iii) dietary supplement intake; and (iv) the Beverage Intake Questionnaire (BEVQ-15) questionnaire to assess the quantity and frequency of sugar-sweetened beverages intake. Participants were provided with an individualized and culturally appropriate dietary plan as an incentive. The presence of hypertension or diabetes mellitus were reported by participants. The presence of hypertension was diagnosed if participants reported having a blood pressure that was ≥130/85 mmHg or if they were under any anti-hypertensive drugs. Participants were considered to have diabetes mellitus if their fasting glucose level was ≥126 mg/dL or their HbA1c level ≥ 6.5 (≥48 mmol/mol), and/or they reported the use of any glucose-lowering drugs.

### 2.3. Anthropometric Data

Anthropometric and laboratory data were also collected. Standing height and body weight were measured in conditions when participants were barefoot and wearing light clothing. The body mass index (BMI) was calculated by dividing the weight (kg) by the height squared (m^2^). In addition, waist circumference was measured using a non-stretchable measuring tape at the level of the umbilicus with a minimal thickness of clothes when the subject was in a standing position and after normal expiration. A bioelectrical impedance analyzer (Omron 511, Tateisi, Japan) was employed to measure lean body mass and visceral fat area of participants as well as appendicular skeletal muscle. We then calculated the skeletal muscle mass index percentage by dividing the total appendicular skeletal mass in (kg) by the body weight (kg). The presence of sarcopenia was detected if the skeletal muscle mass index was ≤37 in males and ≤28 in females; these cut-offs were established as one standard deviation of the mean of young healthy adults. The coexistence of visceral fat obesity—detected through the bioelectrical impedance analyzer—and sarcopenia has been used as a marker that is suggestive of sarcopenic obesity.

### 2.4. MASLD Diagnosis

MASLD cases were referred to the study upon being diagnosed by gastroenterologists. Histological evidence of a fatty liver was detected by gastroenterologists through liver biopsy examination and abdominal ultrasound. Patients with a score of 1 for each of the four histological components (parenchymal brightness, hepatorenal echo contrast, deep beam attenuation, bright vessel walls) were diagnosed as having a fatty liver.

### 2.5. Statistical Analysis

Data were entered and analyzed using SPSS version 23. Descriptive statistics were performed and presented as frequencies and proportions for categorical variables and as means and standard deviation (SD) for continuous variables. ANOVA and chi-square analyses were employed to test significant differences in clinical and biochemical variables where needed.

## 3. Results

Data at baseline was available for 120 patients equally divided between the case and control groups. The demographic and the major clinical, anthropometric, and dietary characteristics of participants were stratified by cases (MASLD) and controls (patients free from MASLD) and are illustrated in Table 1, Table 2, Table 3, Table 4 and Table 5.

### 3.1. Demographic and Clinical Characteristics

The demographic characteristics of participants are present in Table 1. The case and control groups were almost equally divided by age and gender. No significant differences were noted between the case and control groups with respect to smoking status. However, patients diagnosed with MASLD were more likely to have been smoking for a longer duration, notably between 5 to 20 years.

**Table 1:** Demographic characteristics of participants in the case-control groups.

		Control Group		MASLD		*p*-Value
Age in Years (Mean ± SD)		49 ± 11		49 ± 11		0.84
		**N**	**(%)**	**N**	**(%)**	
Age Categories	Young adults	6	(10.0)	6	(10.0)	0.98
	Early Middle-Aged Adults	24	(40.0)	24	(40.0)	
	Late Middle Aged	26	(43.3)	27	(45.0)	
	Elderly	4	(6.7)	3	(5.0)	
Gender	Female	43	(71.7)	43	(71.7)	1.0
	Male	17	(28.3)	17	(28.3)	
Marital status	Single	4	(6.7)	6	(10.0)	0.29
	Married	55	(91.7)	50	(83.3)	
	Divorced	1	(1.7)	4	(6.7)	
Smoking	No	27	(45.0)	28	(46.7)	0.85
	Yes	33	(55.0)	32	(53.3)	
Number of cigarettes (per day)	Less than 5	27	(45.0)	28	(46.7)	0.60
	5–10	0	(0.0)	1	(1.7)	
	10–20	13	(21.7)	9	(15.0)	
	>20	20	(33.3)	22	(36.7)	
Smoking duration (years)	0	27	(45.0)	28	(46.7)	0.44
	<5 years	6	(10.0)	3	(5.0)	
	5–10 years	4	(6.7)	6	(10.0)	
	10–20 years	6	(10.0)	11	(18.3)	
	>20 years	17	(28.3)	12	(20.0)	

The major medical and clinical characteristics of participants are shown in Table 2 respectively. When cases were asked about how they discovered the presence of their liver condition, the majority (61.7%) were diagnosed with liver disease through a liver test that showed abnormality, while a minority were diagnosed during an obesity consultation (11.7%) or by showing digestive problems (5.0%). These cases were not recently diagnosed, as an equal number of cases had been diagnosed with MASLD for less than one year (48.3%) or had had the disease for at least one year (50.0%) from the start of the data collection.

**Table 2:** Medical and clinical characteristics of participants in the case-control groups.

		Control Group		MASLD		*p*-Value
		N	(%)	N	(%)	
Family history	No	11	(18.3)	11	(18.3)	1.00
	Yes	49	(81.7)	49	(81.7)	
Rationale of diagnosis	None	60	(100.0)	13	(21.7)	0.000
	Routine test	0	(0.0)	0	(0.0)	
	Liver test abnormal	0	(0.0)	37	(61.7)	
	Obesity consultation	0	(0.0)	7	(11.7)	
	Family history of MASLD	0	(0.0)	0	(0.0)	
	Digestive symptoms	0	(0.0)	3	(5.0)	
Duration of disease	Recently diagnosed	60	(100.0)	1	(1.7)	0.000
	Less than 1 year	0	(0.0)	29	(48.3)	
	From 1 to 3 year	0	(0.0)	15	(25.0)	
	More than 3 years	0	(0.0)	15	(25.0)	
Current treatment	No	51	(85.0)	22	(36.7)	<0.001
	Yes	9	(15.0)	38	(63.7)	
Type of treatments	No treatment	51	(85.0)	22	(36.7)	<0.001
	Liver diseases	0	(0.0)	3	(5.0)	
	Hypertension	5	(8.3)	5	(8.3)	
	Diabetes	0	(0.0)	5	(8.3)	
	Hypercholesterolemia	2	(3.3)	16	(26.7)	
	Liver diseases and hypertension	2	(3.3)	9	(15.0)	
Past Medical History	No	53	(88.3)	57	(95.0)	0.18
	Yes	7	(11.7)	3	(5.0)	
Hypertension	Absent	48	(80.0)	36	(60.0)	0.017
	Present	12	(20.0)	24	(40.0)	
Diabetes	Absent	60	(100.0)	42	(70.0)	0.000
	Present	0	(0.0)	18	(30.0)	
Other Past Medical History	None	60	(100.0)	58	(96.7)	0.15
	Respiratory and circulatory diseases	0	(0.0)	2	(3.3)	
	Digestive diseases	0	(0.0)	0	(0.0)	
	Cancer	0	(0.0)	0	(0.0)	

Exploring the past medical history, cases were more likely to have hypertension (40.0% vs. 20.0%, *p* = 0.017) and type 2 diabetes mellitus (30.0% vs. 0.0%, *p* = 0.000) compared to those who were free from MASLD. The former group also had a greater proportion of respiratory and circulatory diseases in their past medical history.

### 3.2. Anrthopometric Characteristics

Significant anthropometric differences were observed between the case and control groups (Table 3). The mean weight was significantly higher in the MASLD group (89.98 kg vs. 79.57 kg, *p* < 0.001), along with a greater waist circumference (112.83 cm vs. 100.95 cm, *p* < 0.001). Moreover, a greater proportion of participants in the cases were found to be in the obese category (>30 kg/m²) (86.7% vs. 50.0%, *p* < 0.001) and had a high waist circumference associated with high risk (81.7% vs. 45.0%, *p* < 0.001) compared to the control group. Although not statistically significant, body composition-related differences were noted. Patients with MASLD tended to have a higher fat mass and visceral fat percentage, with a higher proportion falling into the high categories of fat mass (93.4% vs. 91.6%, *p* = 0.070) and visceral fat (75% vs. 45%, *p* = 0.008). Additionally, a greater number of patients in the MASLD group were diagnosed with visceral fat obesity (95.0% vs. 80.0%, *p* = 0.013). Regarding muscle mass differences, cases were more likely to have low muscle mass (78.3% vs. 63.3%, *p* = 0.12). There seems to be a possible association between MASLD and sarcopenia and sarcopenic obesity, as a greater number of participants in the case group had been diagnosed with sarcopenia (90.0% vs. 86.7%, *p* = 0.57) and sarcopenic obesity (88.3% vs. 73.3%, *p* = 0.037) compared to the control group.

**Table 3:** Anthropometric and body composition characteristics of participants in the case-control groups.

		Control Group		MASLD		*p*-Value
Weight (kg) (Mean ± SD)		79.57 ± 11.5		89.98 ± 15.8		0.000
Waist circumference (cm) (Mean ± SD)		100.95 ± 11.37		112.83 ± 12.95		0.000
		**N**	**(%)**	**N**	**(%)**	
Body Mass Index (BMI)		30.05	(5.4)	35.04	(6.4)	0.000
BMI categories	Normal body weight	8	(13.3)	4	(6.7)	0.000
	Overweight	22	(36.7)	4	(6.7)	
	Obesity	30	(50.0)	52	(86.7)	
Waist Circumference Categories	Low risk	9	(15.0)	1	(1.7)	0.000
	Elevated risk	24	(40.0)	10	(16.7)	
	High Risk	27	(45.0)	49	(81.7)	
Fat Mass Categories	Low	1	(1.7)	0	(0.0)	0.070
	Normal	4	(6.7)	4	(6.7)	
	High	8	(13.3)	1	(1.7)	
	Very High	47	(78.3)	55	(91.7)	
Muscle Mass Category	Low	38	(63.3)	47	(78.3)	0.12
	Normal	11	(18.3)	5	(8.3)	
	High	5	(8.3)	2	(3.3)	
	Very high	6	(10.0)	6	(10.0)	
Visceral Fat Categories	Normal	33	(55.0)	15	(25.0)	0.008
	High	25	(41.7)	40	(66.7)	
	Very High	2	(3.3)	4	(8.3)	
Visceral obesity	No	12	(20.0)	3	(5.0)	0.013
	Yes	48	(80.0)	57	(95.0)	
Sarcopenia	No	8	(13.0)	6	(10.0)	0.57
	Yes	52	(86.7)	54	(90.0)	
Sarcopenic-obesity	No	16	(26.7)	7	(11.7)	0.037
	Yes	44	(73.3)	53	(88.3)	

### 3.3. Water and Sugar-Sweetened Beverages Intake

Amongst dietary factors, this study focused on assessing the association between water and sugar-sweetened beverages—including juices, milk, regular soft drinks, artificially sweetened soft drinks, sweetened tea, tea or coffee with cream and/or sugar, tea or black coffee with or without artificial sweetener, alcoholic beverages, and energy drinks—and the risk of MASLD (Table S1). Comparing the two groups, water intake was insufficient among 41.7% of patients with MASLD compared to 43.3 among control group (Table S1).

The consumption patterns of sweetened beverages, specifically 100% fruit juice and sweet juice, appeared quite similar between the cases and controls in terms of frequency and quantity (Table S1). Interestingly, the consumption patterns of whole fat, low-fat, and fat-free milk seemed to be more common among the control group, with control patients consuming these drinks at least two times per week and at least three-quarters of a cup per day.

On the other hand, the consumption patterns of regular soft drinks, sweetened tea, tea or coffee with cream and/or sugar, and tea or black coffee with or without artificial sweetener seemed to be more common among the case group, with cases consuming these drinks at least two times per week and at least three-quarters of a cup per day. Artificially sweetened soft drinks-(diet) were consumed less frequently (<1 per week) among cases (95.0% vs. 91.7%) and in smaller quantities (93.3% vs. 88.3%) compared to the control group.

### 3.4. Alcoholic, Non-Alcoholic Beverages, and Energy Drinks Intake

The consumption patterns of beer, ales, wine, non-alcoholic, or light beer, as well as the quantity of hard liquor, seemed to be more common among the MASLD group, with a higher frequency and quantity of consumption. On the other hand, the frequency pattern of hard liquor and the frequency and quantity of red/white wine and energy and sports drinks were quite similar between the case and control groups. The majority of participants in both groups (around 95–100%) reported consuming these beverages less than one time per week (Table S2). Surprisingly, the consumption of non-alcoholic beers (*p* = 0.03), hard liquor (*p* = 0.021), red or white wine (*p* = 0.02), and energy drinks were found to be positively associated with visceral obesity among patients with MASLD compared to the control group. However, no significant associations were observed with regards to sarcopenia and sarcopenic obesity.

### 3.5. Sarcopenic Obesity, Sarcopenia, and Viseral Fat Obesity

Exploring possible determinants of sarcopenic obesity, sarcopenia, and visceral fat obesity, there is a possible association between age and sarcopenic obesity and visceral fat obesity (*p* = 0.037) among the control group. Patients in the late middle-aged category had a higher probability of having sarcopenic obesity and visceral fat obesity compared to the other age categories. However, this association was not significant for sarcopenia nor in the case group. The association between gender and all three conditions was not statistically significant (*p* > 0.34) among both control and cases. However, there seemed to be a trend towards more cases of visceral fat obesity in females compared to males (Table 4). There were significant associations between the BMI category and all three conditions (*p* < 0.001). Sarcopenic obesity, sarcopenia, and visceral fat obesity were more prevalent in the obesity category compared to the other weight categories among controls. In the cases, the most significant association was seen between the BMI category and visceral fat obesity, with a much higher prevalence in the obesity BMI category (Table 5). The binary logistic regression analyses revealed that no significant determinants of visceral obesity, sarcopenia, and sarcopenic obesity were observed among patients with and without MASLD.

**Table 4:** Associations between age, gender, and BMI with sarcopenic obesity, sarcopenia, and visceral fat obesity in the control group.

		Control														
		Sarcopenic Obesity					Sarcopenia					Visceral Fat Obesity				
		No		Yes		*p*-Value	No		Yes		*p*-Value	No		Yes		*p*-Value
		N	(%)	N	(%)		N	(%)	N	(%)		N	(%)	N	(%)	
Age category	Young adults	4	(25.0)	2	(4.5)	0.037	1	(12.5)	5	(9.6)	0.80	4	(33.3)	2	(4.2)	0.037
	Early middle-aged adults	8	(50.0)	16	(36.4)		4	(50.0)	20	(38.5)		7	(58.3)	17	(35.4)	
	Late middle aged	3	(18.8)	23	(52.3)		3	(37.5)	23	(44.2)		0	(0.0)	26	(54.2)	
	Elderly	1	(6.3)	3	(6.8)		0	(0.0)	4	(7.7)		1	(8.3)	3	(6.3)	
Gender	Female	10	(62.5)	33	(75.0)	0.34	5	(62.5)	38	(73.1)	0.57	6	(50.0)	37	(77.1)	0.34
	Male	6	(37.5)	11	(25.0)		3	(37.5)	14	(26.9)		6	(50.0)	11	(22.9)	
BMI category	Normal body weight	6	(37.5)	2	(4.5)	0.000	4	(50.0)	4	(7.7)	0.001	5	(41.7)	3	(6.3)	0.000
	Overweight	8	(50.0)	14	(31.8)		4	(50.0)	18	(34.6)		5	(41.7)	17	(35.4)	
	Obesity	2	(12.5)	28	(63.6)		0	(0.0)	30	(57.7)		2	(16.7)	28	(58.3)	

**Table 5:** Associations between age, gender, and BMI with sarcopenic obesity, sarcopenia, and visceral fat obesity in the case group.

		Cases														
		Sarcopenic Obesity					Sarcopenia					Visceral Fat Obesity				
		No		Yes		*p*-Value	No		Yes		*p*-Value	No		Yes		*p*-Value
		N	(%)	N	(%)		N	(%)	N	(%)		N	(%)	N	(%)	
Age category	Young adults	1	(14.3)	5	(9.4)	0.90	1	(16.7)	5	(9.3)	0.80	1	(33.3)	5	(8.8)	0.57
	Early middle-aged adults	3	(42.9)	21	(39.6)		3	(50.0)	21	(38.9)		1	(33.3)	23	(40.4)	
	Late middle aged	3	(42.9)	24	(45.3)		2	(33.3)	25	(46.3)		1	(33.3)	26	(45.6)	
	Elderly	0	(0.0)	3	(5.7)		0	(0.0)	3	(5.6)		0	(0.0)	3	(5.3)	
Gender	Female	4	(57.1)	39	(73.6)	0.36	4	(66.7)	39	(72.2)	0.77	0	(0.0)	43	(75.4)	0.055
	Male	3	(42.9)	14	(26.4)		2	(33.3)	15	(27.8)		3	(100.0)	14	(24.6)	
BMI category	Normal body weight	2	(28.6)	2	(3.8)	0.027	2	(33.3)	2	(3.7)	0.010	2	(66.7)	2	(3.5)	0.000
	Overweight	1	(14.3)	3	(5.7)		1	(16.7)	3	(5.6)		0	(0.0)	4	(7.0)	
	Obesity	4	(57.1)	48	(90.6)		3	(50.0)	49	(90.7)		1	(33.3)	51	(89.5)	

## 4. Discussion

To the best of our knowledge, this was the first study in Lebanon and in the MENA region that explored possible differences in medical, clinical, anthropometric, and dietary characteristics between subjects with or without MASLD among the Lebanese population through a case-control design. Additionally, the study delved into the possible determinants of visceral fat obesity, sarcopenia, and sarcopenic obesity.

Findings from the present study demonstrated a clear association between the presence of MASLD and obesity, hypertension, and diabetes. The pattern of obesity and the percentage and type of body fat found in the cases compared to the control group were consistent with findings from previous prospective cohort studies conducted in the United States, and in European and Asian settings that consistently uncovered a significantly higher BMI and body fat percentage and specifically, visceral body fat in patients with MASLD [30,31,32,33,34,35]. The association between obesity and MASLD is thought to be mediated by several mechanisms. One of the most important mechanisms is the effect of obesity on insulin resistance. Excess adipose tissue can lead to insulin resistance, which, in turn, can promote the development of MASLD. In addition, obesity is associated with chronic low-grade inflammation, which can also contribute to the development of MASLD [35].

The waist was also closely associated with MASLD as anticipated in previous studies that have highlighted the relationships between alterations in central adiposity and the occurrence of MASLD [34,36].

The observed average of significant differences in blood pressure among cases compared to the control group in this study suggested that hypertension is also associated with MASLD. The average significance was explained by a review that critically discussed the body of clinical evidence that supported the existence of a bi-directional relationship between MASLD and metabolic syndrome components which showed that hypertension plays a role in MASLD development, yet, with a small effect on the risk of incident MASLD than other components of metabolic syndrome [37]. Moreover, in the present study, patients with MASLD displayed a higher prevalence of diabetes, which also corroborated the body of evidence and reviews showing a high prevalence of advanced fibrosis, hepatic steatosis, and liver-related complications among patients with type 2 diabetes mellitus, whether they were obese or not [38,39,40,41,42].

**Figure 1:**
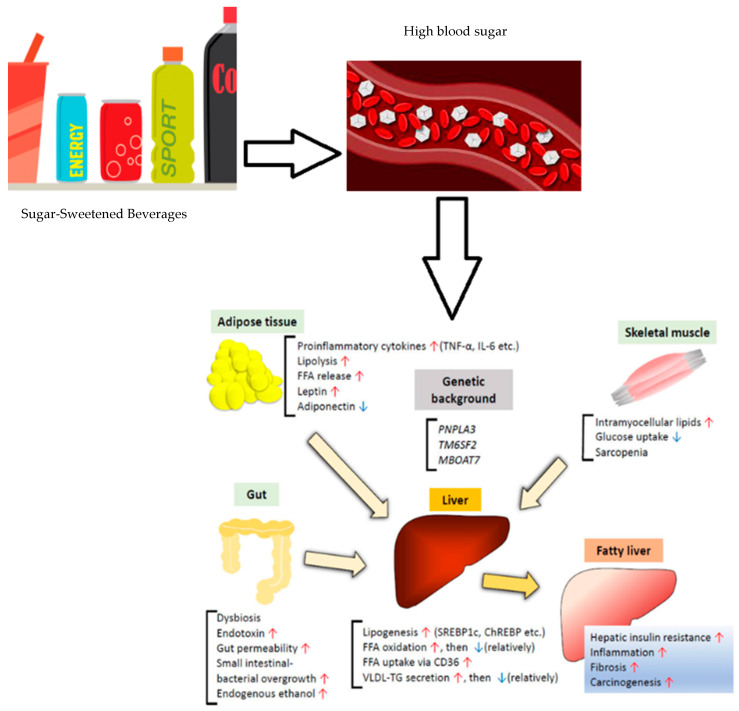
Effect of consumption of sugar-sweetened beverages on MASLD. Adapted from Sakurai et al., 2021 [56]. Red arrows indicate increase in the actions and blue arrows indicate its decrease.

On the other hand, a non-significant relationship was observed between smoking and a non-active lifestyle and the presence of MASLD. The effect of environmental factors is not very well established in the literature yet as there is only limited evidence showing an inverse significant association between physical activity and smoking and the prevalence of MASLD [28,43,44]. Similar to our findings, studies examining the relationship between sarcopenic obesity and MASLD, found a higher proportion of MASLD patients having sarcopenic obesity, which is in line with the findings of the current study where MASLD cases were more likely to have sarcopenic obesity compared to the control group [28,45]. Multivariate regression analysis conducted in these studies highlighted the significant association of MASLD with sarcopenic obesity, independently of age, gender, or other metabolic confounders showing that patients with MASLD have a more than 3-fold chance of having sarcopenic obesity. This suggests that MASLD might be a risk factor for sarcopenic obesity [27,45]. Further research needs to be conducted to explore whether sarcopenic obesity is a cause or a consequence of MASLD or other liver diseases. The relationship between sarcopenia and MASLD has been the subject of recent studies and discussions. This association is especially interesting in patients with the co-exitance of both obesity and sarcopenia as high amounts of visceral fat have been described to be a predisposing factor for MASLD, yet, this study did not find sarcopenia and the diagnosis of the disease, however, there was a mild association spotted between sarcopenic obesity and the presence of MASLD. A body of evidence concluding an inverse insulin resistance and obesity-independent correlation between skeletal muscle index and MASLD in cross-sectional and in longitudinal studies where the appendicular skeletal muscle mass decreased over time and showed significantly increasing odds for incident MASLD [46,47,48,49]. This connection was also a persistent event after the adjustment of age, gender, BMI, hypertension, diabetes, and smoking status [50]. We finally focused on the relationship between water and sugar-sweetened beverages (SSBs) among MASLD cases, as the dietary aspect of this study, finding a higher consumption of SSBs including regular soft drinks, sweetened tea, tea or coffee with cream and/or sugar or with/without artificial sweetener and a lower consumption of milk and water amongst among the cases. The results were in line with a body of literature showing a clear consensus when it came to the consumption of SSBs with the risk of MASLD with a 1.39–1.49-fold increase in the odds [51,52] and with the increase in visceral fats when compared to milk and water [53,54]. One of the major modifiable risk factors for MASLD is the consumption of sugar-sweetened beverages, because they are a rapidly consumed source of calories without leading to satiety which leads to gaining weight. Gaining weight by as little as 7 to 11 pounds and consuming diets high in sugar or calories can predict MASLD. This is because a high intake of these foods results in increased fat accumulation in the liver, insulin resistance, and inflammation [55]. Figure 1 shows the impact of consumption of sweetened beverages on MASLD.

As for alcohol consumption, no major significant associations were observed between MASLD and the consumption of non-alcoholic and alcoholic beverages. This lack of significant association could potentially be attributed to the cultural context prevalent in Arab countries, where Islamic norms prohibit the consumption of alcohol. This prohibition could have limited the diversity of the sample, potentially influencing the ability to uncover accurate associations. In our subgroup analyses performed to assess the associations between age, gender, and BMI with sarcopenic obesity, sarcopenia, and visceral fat obesity, the positive association with a higher BMI and all of the three conditions persisted consistently, even when the data was stratified by case and control groups. Moreover, a discernible trend emerged in which middle-aged participants displayed a heightened likelihood of experiencing sarcopenic obesity and visceral fat obesity. This pattern aligned with findings from previous literature, reinforcing the validity of our observations [57,58]. Some limitations and bias were present in the study. The sample of the study population was not epidemiologically representative of the Lebanese population, nonetheless, the outcomes retain relevance as they mark the pioneering case-control investigation in Lebanon delving into the clinical attributes of individuals afflicted with MASLD. This inquiry also scrutinized the factors that links with visceral fat obesity sarcopenia, and sarcopenic obesity. It is essential to acknowledge that recall biases could have impacted the results, given that self-reported information on factors such as smoking status, alcohol consumption, and physical activity was utilized. However, diligent efforts were undertaken to mitigate this potential bias. Interviewers underwent comprehensive training to ensure consistent and accurate data collection procedures, aiming to minimize the influence of recall bias. Furthermore, due to lack of funding, our research team could not conduct laboratory analysis, metabolism analysis, liver studies, or any histological analysis to investigate the change in metabolic profile between both study groups.

## 5. Conclusions

Our findings indicated a notable correlation between the presence of MASLD and obesity, hypertension, and diabetes, along with a significant connection between higher BMI and visceral obesity, sarcopenia, and sarcopenic obesity. Lastly, in exploring dietary aspects, we observed a higher consumption of SSBs among MASLD cases, alongside reduced milk and water consumption among cases. This study shed light on the health attributes and diets of the Lebanese population with liver diseases and suggests more research in this area and in a more ethnically diverse population.

## Data Availability

Data are contained within the article and Supplementary Materials.

## Supplementary Materials

The following supporting information can be downloaded at: https://www.mdpi.com/article/10.3390/healthcare12050591/s1, Table S1. Water and sugary-sweetened beverages consumption among case-control groups. Table S2. Intake of alcoholic, non-alcoholic beverages, and energy drinks among case-control groups.
