# Supplementary material for: Influence of Sugar-Sweetened Beverages Intake on Sarcopenic Obesity, Visceral Obesity, and Sarcopenia in Lebanese Patients with MASLD: A Case-Control Study
Source: Healthcare (Basel). 2024 Mar 5;12(5):591. doi: 10.3390/healthcare12050591 (PMC10931226; doi:10.3390/healthcare12050591)
Supplement: Supplementary file 1 [file healthcare-12-00591-s001.zip › healthcare-2820043-supplementary.pdf]

Table S1. Water and sugary-sweetened beverages consumption among case-control groups.

		Control Group		MASLD	
		N	(%)	N	(%)
**Water** Frequency	< 1 per /week	1	(1.7)	2	(3.3)
	1 /week	2	(3.3)	0	(0.0)
	2 to 3 /week	3	(5.0)	4	(6.7)
	4 to 6 /week	3	(5.0)	2	(3.3)
	1 /day	3	(5.0)	2	(3.3)
	At least 2 /day	5	(8.3)	2	(3.3)
	At least 3 /day	43	(71.7)	48	(80.0)
**Water** Quantity (based on dietary guidelines)	Insufficient	26	(43.3)	25	(41.7)
	Sufficient	34	(56.7)	35	(58.3)
**100% Fruit juice** Frequency	< 1 per /week	39	(65.0)	38	(63.3)
	1 /week	4	(6.7)	3	(5.0)
	2 to 3 /week	11	(18.3)	14	(23.3)
	4 to 6 /week	4	(6.7)	1	(1.7)
	1 /day	1	(1.7)	3	(5.0)
	At least 2 /day	1	(1.7)	0	(0.0)
	At least 3 /day	0	(0.0)	1	(1.7)
**100% Fruit juice** Quantity	0	30	(50.0)	29	(48.3)
	3/4 cup	0	(0.0)	0	(0.0)
	1 cup	27	(45.0)	26	(43.3)
	1/2 cup	0	(0.0)	3	(5.0)
	2 cups	1	(1.7)	1	(1.7)
	21/2 cup	2	(3.3)	1	(1.7)
	3 cups	0	(0.0)	0	(0.0)
**Sweet Juice** Frequency	< 1 per /week	48	(80.0)	47	(78.3)
	1 /week	5	(8.3)	5	(8.3)
	2 to 3 /week	3	(5.0)	5	(8.3)
	4 to 6 /week	0	(0.0)	3	(5.0)
	1 /day	2	(3.3)	0	(0.0)
	At least 2 /day	0	(0.0)	0	(0.0)
	At least 3 /day	2	(3.3)	0	(0.0)
**Sweet Juice** Quantity	0	53	(88.3)	52	(86.7)
	1 cup	4	(6.7)	5	(8.3)

	2 cups	1	(1.7)	1	(1.7)
	4 cups	0	(0.0)	2	(3.3)
	6 cups	2	(3.3)	0	(0.0)
**Whole fat Milk** Frequency	< 1 per /week	48	(80.0)	50	(83.3)
	1 /week	0	(0.0)	3	(5.0)
	2 to 3 /week	6	(10.0)	2	(3.3)
	4 to 6 /week	2	(3.3)	2	(3.3)
	1 /day	0	(0.0)	2	(3.3)
	At least 2 /day	0	(0.0)	0	(0.0)
	At least 3 /day	4	(6.7)	1	(1.7)
**Whole fat Milk** Quantity	0	38	(63.3)	38	(63.3)
	3/4 cup	1	(1.7)	2	(3.3)
	1 cup	20	(33.3)	17	(28.3)
	1/2 cup	0	(0.0)	1	(1.7)
	2 cups	1	(1.7)	2	(3.3)
	21/2 cup	0	(0.0)	0	(0.0)
**Reduced Milk** Frequency	< 1 per /week	56	(93.3)	57	(95.0)
	1 /week	1	(1.7)	1	(1.7)
	2 to 3 /week	2	(3.3)	0	(0.0)
	4 to 6 /week	0	(0.0)	0	(0.0)
	1 /day	1	(1.7)	2	(3.3)
	At least 2 /day	0	(0.0)	0	(0.0)
	At least 3 /day	0	(0.0)	0	(0.0)
**Reduced Milk** Quantity	0	56	(93.3)	56	(93.3)
	3/4 cup	0	(0.0)	2	(3.3)
	1 cup	4	(6.7)	2	(3.3)
	1/2 cup	0	(0.0)	0	(0.0)
	2 cups	0	(0.0)	0	(0.0)
	21/2 cup	0	(0.0)	0	(0.0)
**Low fat-free fat Milk** Frequency	< 1 per /week	56	(93.3)	58	(96.7)
	1 /week	1	(1.7)	0	(0.0)
	2 to 3 /week	2	(3.3)	1	(1.7)
	4 to 6 /week	0	(0.0)	0	(0.0)
	1 /day	0	(0.0)	1	(1.7)
	At least 2 /day	0	(0.0)	0	(0.0)
	At least 3 /day	1	(1.7)	0	(0.0)
**Low fat-free fat Milk** Quantity	0	54	(90.0)	56	(93.3)
	3/4 cup	0	(0.0)	2	(3.3)
	1 cup	5	(8.3)	2	(3.3)

	1/2 cup	0	(0.0)	0	(0.0)
	2 cups	0	(0.0)	0	(0.0)
	21/2 cup	1	(1.7)	0	(0.0)
**Regular soft Drink** Frequency	< 1 per /week	46	(76.7)	41	(68.3)
	1 /week	4	(6.7)	6	(10.0)
	2 to 3 /week	2	(3.3)	4	(6.7)
	4 to 6 /week	2	(3.3)	6	(10.0)
	1 /day	1	(1.7)	1	(1.7)
	At least 2 /day	1	(1.7)	0	(0.0)
	At least 3 /day	4	(6.7)	2	(3.3)
**Regular soft Drink** Quantity	0	38	(63.3)	28	(46.7)
	3/4 cup	1	(1.7)	2	(3.3)
	1 cup	14	(23.3)	21	(35.0)
	1/2 cup	1	(1.7)	2	(3.3)
	2 cups	5	(8.3)	6	(10.0)
	21/2 cup	1	(1.7)	1	(1.7)
**Soft drink- Artificially Sweetened (Diet)** Frequency	< 1 per /week	55	(91.7)	57	(95.0)
	1 /week	2	(3.3)	1	(1.7)
	2 to 3 /week	1	(1.7)	1	(1.7)
	4 to 6 /week	0	(0.0)	0	(0.0)
	1 /day	1	(1.7)	0	(0.0)
	At least 2 /day	0	(0.0)	0	(0.0)
	At least 3 /day	1	(1.7)	1	(1.7)
**Soft drink- Artificially Sweetened (Diet)** Quantity	0	53	(88.3)	56	(93.3)
	3/4 cup	1	(1.7)	1	(1.7)
	1 cup	5	(8.3)	2	(3.3)
	1/2 cup	0	(0.0)	1	(1.7)
	2 cups	0	(0.0)	0	(0.0)
	21/2 cup	1	(1.7)	0	(0.0)
**Sweetened Tea** Frequency	< 1 per /week	22	(36.7)	16	(26.7)
	1 /week	2	(3.3)	2	(3.3)
	2 to 3 /week	2	(3.3)	0	(0.0)
	4 to 6 /week	3	(5.0)	2	(3.3)
	1 /day	5	(8.3)	4	(6.7)
	At least 2 /day	4	(6.7)	1	(1.7)
	At least 3 /day	22	(36.7)	35	(58.3)
**Sweetened Tea** Quantity	0	16	(26.7)	14	(23.3)
	3/4 cup	0	(0.0)	0	(0.0)
	1 cup	29	(48.3)	17	(28.3)

	1/2 cup	1	(1.7)	0	(0.0)
	2 cups	8	(13.3)	21	(35.0)
	21/2 cup	6	(10.0)	8	(13.3)
**Tea or Coffee with cream and/or sugar** Frequency	< 1 per /week	40	(66.7)	37	(61.7)
	1 /week	0	(0.0)	2	(3.3)
	2 to 3 /week	1	(1.7)	2	(3.3)
	4 to 6 /week	1	(1.7)	1	(1.7)
	1 /day	1	(1.7)	0	(0.0)
	At least 2 /day	2	(3.3)	1	(1.7)
	At least 3 /day	15	(25.0)	17	(28.3)
**Tea or Coffee with cream and/or sugar** Quantity	0	36	(60.0)	33	(55.0)
	3/4 cup	2	(3.3)	1	(1.7)
	1 cup	16	(26.7)	18	(30.0)
	1/2 cup	0	(0.0)	0	(0.0)
	2 cups	5	(8.3)	3	(5.0)
	21/2 cup	1	(1.7)	5	(8.3)
**Tea or Coffee black with or without artificial sweetener** Frequency	< 1 per /week	35	(58.3)	22	(36.7)
	1 /week	0	(0.0)	3	(5.0)
	2 to 3 /week	1	(1.7)	6	(10.0)
	4 to 6 /week	1	(1.7)	5	(8.3)
	1 /day	0	(0.0)	1	(1.7)
	At least 2 /day	2	(3.3)	0	(0.0)
	At least 3 /day	21	(35.0)	23	(38.3)
**Tea or Coffee black with or without artificial sweetener** Quantity	0	30	(50.0)	17	(28.3)
	3/4 cup	1	(1.7)	1	(1.7)
	1 cup	12	(20.0)	18	(30.0)
	1/2 cup	1	(1.7)	0	(0.0)
	2 cups	2	(3.3)	12	(20.0)
	21/2 cup	14	(23.3)	12	(20.0)

Table S2. Intake of alcoholic, non-alcoholic beverages, and energy drinks among case-contol groups.

		**Control Group**		**MASLD**	
		**N**	**(%)**	**N**	**(%)**
**Beer, Ales, Wine, Non-**	< 1 per /week	60	(100.0)	57	(95.0)
	1 /week	0	(0.0)	2	(3.3)
	2 to 3 /week	0	(0.0)	0	(0.0)

**alcoholic or light Beer** Frequency	4 to 6 /week	0	(0.0)	0	(0.0)
	1 /day	0	(0.0)	0	(0.0)
	At least 2 /day	0	(0.0)	0	(0.0)
	At least 3 /day	0	(0.0)	1	(1.7)
**Beer, Ales, Wine, Non-alcoholic or light Beer** Quantity	0	60	(100.0)	56	(93.3)
	3/4 cup	0	(0.0)	2	(3.3)
	1 cup	0	(0.0)	1	(1.7)
	1/2 cup	0	(0.0)	1	(1.7)
	2 cups	0	(0.0)	0	(0.0)
	21/2 cup	0	(0.0)	0	(0.0)
**Hard Liquor** Frequency	< 1 per /week	60	(100.0)	60	(100.0)
	1 /week	0	(0.0)	0	(0.0)
	2 to 3 /week	0	(0.0)	0	(0.0)
	4 to 6 /week	0	(0.0)	0	(0.0)
	1 /day	0	(0.0)	0	(0.0)
	At least 2 /day	0	(0.0)	0	(0.0)
	At least 3 /day	0	(0.0)	0	(0.0)
**Hard Liquor** Quantity	0	60	(100.0)	57	(95.0)
	3/4 cup	0	(0.0)	3	(5.0)
	1 cup	0	(0.0)	0	(0.0)
	1/2 cup	0	(0.0)	0	(0.0)
	2 cups	0	(0.0)	0	(0.0)
	21/2 cup	0	(0.0)	0	(0.0)
**Wine Red/White** Frequency	< 1 per /week	60	(100.0)	60	(100.0)
	1 /week	0	(0.0)	0	(0.0)
	2 to 3 /week	0	(0.0)	0	(0.0)
	4 to 6 /week	0	(0.0)	0	(0.0)
	1 /day	0	(0.0)	0	(0.0)
	At least 2 /day	0	(0.0)	0	(0.0)
	At least 3 /day	0	(0.0)	0	(0.0)
**Wine Red/White** Quantity	0	58	(96.7)	57	(95.0)
	3/4 cup	2	(3.3)	3	(5.0)
	1 cup	0	(0.0)	0	(0.0)
	1/2 cup	0	(0.0)	0	(0.0)
	2 cups	0	(0.0)	0	(0.0)
	21/2 cup	0	(0.0)	0	(0.0)
**Energy & Sports drinks** Frequency	< 1 per /week	60	(100.0)	60	(100.0)
	1 /week	0	(0.0)	0	(0.0)
	2 to 3 /week	0	(0.0)	0	(0.0)

	4 to 6 /week	0	(0.0)	0	(0.0)
	1 /day	0	(0.0)	0	(0.0)
	At least 2 /day	0	(0.0)	0	(0.0)
	At least 3 /day	0	(0.0)	0	(0.0)
**Energy & Sports drinks** Quantity	0	58	(96.7)	57	(95.0)
	3/4 cup	1	(1.7)	3	(5.0)
	1 cup	0	(0.0)	0	(0.0)
	1/2 cup	0	(0.0)	0	(0.0)
	2 cups	1	(1.7)	0	(0.0)
	21/2 cup	0	(0.0)	0	(0.0)
